# Special Issue “Genetic Modifiers of Hemoglobinopathies: Recent Advances and Future Directions”

**DOI:** 10.3390/ijms27041890

**Published:** 2026-02-16

**Authors:** Carsten W. Lederer, Alex E. Felice, Petros Kountouris

**Affiliations:** 1 Blood Disorder Genetics & Thalassemia, The Cyprus Institute of Neurology & Genetics, 6 Iroon Avenue, Ayios Dometios, Nicosia 2371, Cyprus; 2 Thalassaemia Testing and Haemoglobin Research Laboratory, University of Malta, and Thalassaemia Clinic, Mater Dei Hospital, MSD2090 Msida, Malta

## 1. Introduction

Hemoglobinopathies are monogenic disorders that primarily affect erythrocyte biology but show high phenotypic diversity in the number of associated disease phenomena and overall disease severity. This diversity partly reflects distinct pathogenic variants in the α- and β-globin gene clusters, which cause qualitative disorders such as sickle cell anemia or quantitative disorders such as thalassemias. Pathological changes in main adult hemoglobin (HbA, α_2_β_2_) levels, α-to-β globin balance, and downstream phenotypes are further shaped by variants in globin paralogs and non-globin loci acting as genetic disease modifiers (GDMs). This is epitomized by variants in repressors of the β-like fetal γ-globin gene, which already serve as the basis of curative advanced therapies for both sickle cell disease (SCD) and β-thalassemia. The comprehensive characterization of GDMs across pathological phenomena and diverse patient populations is thus critical for prognosis, treatment choices, and therapy development for hemoglobinopathies. In this Special Issue of the *International Journal of Molecular Sciences*, we therefore set out to establish the status quo of our current knowledge on genetic modifiers in hemoglobinopathies, to identify gaps in our understanding, and to outline the most promising paths for future research. We anticipate that the resulting articles and insights will encourage and guide corresponding international collaborative efforts, such as those by the International Hemoglobinopathy Research Network (INHERENT), which addresses prior limitations in sample size, diversity, and statistical power through large-scale, multi-ethnic, genome-wide association studies (GWASs) in hemoglobinopathy cohorts [1,2].

## 2. Articles in the Special Issue

A key area of GDM action is direct effects on globin and hemoglobin expression, various facets of which were investigated by three different reviews in this Special Issue. The systematic review by Stephanou et al. comprehensively characterizes variants and pathways associated with fetal hemoglobin (HbF, α_2_γ_2_) expression and HbF-positive cells, properties that have a profound impact on disease severity and phenomena across all articles and topics discussed in this Special Issue (Contribution 1). Meanwhile, Diamantidis et al. focus on the clinical relevance in β-thalassemia and SCD of factors that determine HbF and HbA levels (Contribution 2). The article integrates evidence on transcriptional regulators, chromatin modifiers, and epigenetic factors that modulate globin gene expression, and discusses pharmacological and genome editing strategies aimed at therapeutic HbF induction. Traeger-Synodinos et al., meanwhile, extend the discussion on locus-specific modifier mechanisms across α- and β-like globins, assessing how the differential expression of α-globin genes and related regulatory networks alters phenotype severity in β-thalassemia and SCD (Contribution 3). The resulting interplay of structural and regulatory variants and effects on globin chain balance further add to the complexity already apparent for γ-globin–related modifiers. This is amplified by Harteveld et al. with their review of *SUPT5H* loss-of-function variants and their effect on clinical severity in β-thalassemia patients (Contribution 4). Their analysis of the SUPT5H transcription factor as a plausible contributor to globin gene regulation and erythroid differentiation exemplifies the discovery of gene-specific modifiers with direct mechanistic implications.

Globin deregulation, with ensuing defective myeloid erythropoiesis, peripheral lysis or sickling of erythrocytes, and corresponding anemia, iron overload, and cardiovascular defects, then determines additional organ-specific pathologies. These are, in turn, influenced by myriad genetic factors. In this context, Labarque and Okocha systematically review GDMs associated with nephropathy in SCD patients, integrating evidence from GWASs and candidate gene studies to highlight loci implicated in albuminuria, glomerular hyperfiltration, and net renal function decline. By addressing protective α-thalassemia variants, risk-increasing APOL1 variants, and other kidney-related loci, the review highlights the age-dependent heterogeneity of GDM effects and the need for functional validation (Contribution 5). A scoping review by Oni et al. investigates GDMs associated with stroke, both ischemic and hemorrhagic, as well as silent white matter changes, touching on severe complications that are all the more relevant for their relatively high prevalence in SCD patients (Contribution 6). A scoping review by Sophocleous et al. instead investigates GDMs associated with the frequency and severity of pain phenomena and vaso-occlusive crises as a hallmark complication of SCD. By cataloguing GDMs in inflammatory, coagulation, nitric oxide, and drug response pathways, the review identifies candidate GDMs, pathway interactions, and corresponding subjects and improvements for future studies (Contribution 7). In further reviews, Chatzidavid et al. turn to pulmonary hypertension in SCD and the effect of GDMs on related pathologies (Contribution 8), while Gambari et al. summarize pharmacogenomic insights for drugs employed in the treatment of β-thalassemia or SCD (Contribution 9). Finally, Evangelides et al. investigate the effect of excess iron in hemoglobinopathies on a range of endocrinopathies, summarizing findings for the role of GDMs on erythropoiesis and iron metabolism, with emphasis on the importance of oxidative stress pathways and erythrocyte membrane stability (Contribution 10). Together, these articles cover diverse GDMs and methodologies, from candidate gene studies to GWASs, underscoring the need for geographically diverse cohorts to identify robust genotype–phenotype correlations and clinically relevant biomarkers and therapeutic targets.

## 3. Beyond the Special Issue

Our dear co-author and co-editor, Prof. Alex E. Felice, sadly passed away during the preparation of this editorial. A pioneer of thalassemia genetics in Malta [3], he would have been at pains to point out that high-throughput GDM identification only yields actionable insights with full functional characterization. He would have emphasized the overriding distinction between hemotropic GDMs, which have wider effects by acting on blood lineage cells from HSC commitment to erythrocyte turnover, and somatotropic GDMs, which primarily affect secondary tissues and organs. Many GDM-influenced hemoglobinopathy phenotypes fall outside the scope of this Special Issue but merit mention here as priorities for future investigation.

Erythropoiesis itself is of central importance in both SCD and thalassemia as erythroid disorders and offers extensive data on GDMs, although it is not the focus of a dedicated review in this Special Issue and is instead addressed across several articles (Contributions 1–3, 9). Also of importance is the topic of intramedullary and peripheral inflammation, with known association of a plethora of genes and a diversity of effects that range from stress erythropoiesis to accelerated erythrocyte turnover [4]. Osteoporosis likewise represents a major source of morbidity in SCD and thalassemia, yet a comprehensive collection of associated GDMs in this disease context is still lacking [5,6]. The same is true for ulcers, although in particular for the thalassemias, making the association between GDMs and ulcers may rely on research outside the specific disease context and on related phenotypes, such as angiogenesis [7,8]. Co-inheritance of Gilbert syndrome is also relevant in both SCD and thalassemia, as are other disorders that reduce the capacity to detoxify or eliminate disease-related metabolites such as bilirubin [9]. Distinct clinical features of SCD and thalassemia imply partly distinct sets of associated loci and GDMs. For instance, retinopathy is a major cause of blindness in SCD and, as the most prevalent vascular complication with numerous associated GDMs, would warrant a separate review. Likewise, splenic complications are of vital concern in SCD, where acute splenic sequestration crises because of hypersplenism [10] and acute sepsis because of asplenia [11] may both lead to life-threatening emergencies.

It turns out that GDMs remain poorly characterized for splenic complications, as for many other clinically critical phenomena in hemoglobinopathies. As a pointer to future work by INHERENT and other systematic GDM discovery efforts, this is an important insight in and of itself. Future GDM studies should therefore address additional disease phenotypes using expanded, diverse cohorts and updated study designs. Priorities include multi-omics integration, epigenetic studies of stress and differentiation responses, the assessment of GDM effects on therapy response, the development of GDM-based treatment stratification algorithms, and expansion to diverse populations to ensure their representation in risk modeling.

## 4. Conclusions

The Special Issue on genetic modifiers of hemoglobinopathies consolidates current advances and highlights the complexity of genotype–phenotype relationships beyond primary globin mutations. Despite progress in identifying candidate loci and regulatory pathways, clinical translation of such knowledge remains limited, and many disease features and analytical approaches are still unexplored. Future work must strive for robust multi-ethnic data and functional validation to link discoveries with mechanistic understanding and clinical actionability. Building on the insights compiled in this Special Issue, these steps and integrated research networks such as INHERENT can advance precision medicine in hemoglobinopathy diagnosis, prognosis, and therapy, supporting informed treatment decisions and equitable care.

## Abbreviations

The following abbreviations are used in this manuscript:

GDM	genetic disease modifier
GWAS	genome-wide association study
HbA	main adult hemoglobin (α_2_β_2_)
HbF	fetal hemoglobin (α_2_γ_2_)
HSC	hematopoietic stem cells
SCD	sickle cell disease
